# Pharmacokinetics of Immediate and Sustained Release Cephalexin Administered by Different Routes to Llamas (*Lama glama*)

**DOI:** 10.1155/2016/4621039

**Published:** 2016-03-09

**Authors:** Verónica Kreil, Luis Ambros, Ana Paula Prados, Lisa Tarragona, Agustina Monfrinotti, Guillermo Bramuglia, Marcela Rebuelto

**Affiliations:** ^1^Farmacología, Facultad de Ciencias Veterinarias, Universidad de Buenos Aires, Chorroarín 280, 1427 Ciudad Autónoma de Buenos Aires, Argentina; ^2^Farmacología, Facultad de Farmacia y Bioquímica, Universidad de Buenos Aires, Junín 1100, 1425 Ciudad Autónoma de Buenos Aires, Argentina

## Abstract

We investigate the pharmacokinetics of two different cephalexin formulations administered to llamas by the intravenous (IV), intramuscular (IM), and subcutaneous (SC) routes, the minimum inhibitory concentration (MIC) of cephalexin against some* Escherichia coli* and staphylococci isolated from llamas, and we apply the PK/PD modelling approach, so that effective dosage recommendations for this species could be made. Six llamas received immediate (10 mg/kg, IV, IM, and SC) and sustained (8 mg/kg IM, SC) release cephalexin. Pharmacokinetic parameters were calculated by noncompartmental approach. Immediate release SC administration produced a significantly longer elimination half-life as compared with the IV and IM administration (1.3 ± 0.2 versus 0.6 ± 0.1 and 0.6 ± 0.1 h, resp.) and higher mean absorption time as compared with the IM administration (1.7 ± 0.5 versus 0.6 ± 0.4 h). Absolute bioavailability was in the range of 72–89% for both formulations and routes of administration. Cephalexin MIC_90_ values against staphylococci and* E. coli *were 1.0 and 8.0 *μ*g/mL, respectively. Our results show that the immediate release formulation (10 mg/kg) would be effective for treating staphylococcal infections administered every 8 h (IM) or 12 h (SC), whereas the sustained release formulation (8 mg/kg) would require the IM or SC administration every 12 or 24 h, respectively.

## 1. Introduction

The administration of drugs with therapeutic purposes must be done selecting a dosage regimen both effective and safe. For the anti-infective therapy, a close relationship between plasma concentrations and antibacterial activity of the chosen antibiotic has been previously demonstrated by PK/PD modelling, and the optimal dosage regimen has been determined for several antibiotics and species using a surrogate index of clinical outcome; that is, for beta-lactam antibiotics, the time for which plasma concentrations is above the minimum inhibitory concentration (MIC) of the invading pathogen (*T* > MIC) [1]. However, pharmacokinetic and MIC data about South American camelids as llamas (*Lama glama*) are scarce, and veterinarians estimate the dosage regimen based on information obtained from other species, usually ruminants. This extrapolation may result in ineffective therapies, mainly due to the unique interspecies pharmacokinetic differences in drug transport across membranes, protein binding, and drug metabolism and excretion [1].

Cephalexin is a beta-lactam antibiotic with good activity against Gram-positive bacteria, such as* Staphylococcus* spp. and* Streptococcus* spp., and low activity against some Enterobacteriaceae as* Escherichia coli*. Cephalexin is frequently used in veterinary medicine due to its high bactericidal efficacy, low cost, and lack of toxicity. Cephalexin may be administered by the oral or parenteral routes, and currently available commercial formulations may provide an immediate or a sustained release of the drug, thus prolonging the duration of the antibacterial activity (long-acting formulations). Cephalexin pharmacokinetics has been described in several domestic species, such as dogs [2, 3], horses [4], and ruminants [5–11]; however, pharmacokinetic reports on conventional and long-acting cephalexin pharmacokinetics in llama are lacking. The purposes of this study were to investigate the pharmacokinetics of cephalexin formulated as an immediate and sustained release commercial formulation when administered to healthy adult llamas as single bolus by the intravenous (IV), intramuscular (IM), and subcutaneous (SC) routes, to determine the MIC of cephalexin against some* Escherichia coli* and coagulase-positive staphylococci isolated from llamas, and to apply the PK/PD modelling approach, so that effective dosage recommendations for this species can be made.

## 2. Materials and Methods

### 2.1. Animals

Adult llamas, property of the Facultad de Ciencias Veterinarias, Universidad de Buenos Aires, were used in this study. Animals were determined to be clinically healthy based on history, physical examination, and haematological evaluation. None of them had been treated with antibiotics for one month prior to the trial. Llamas were housed in a shed with access to concentrate, green food, and water* ad libitum*. For dose calculation llamas were weighed the day of each treatment. Animal procedures were approved by the Institutional Animal Care and Use Committee of Facultad de Ciencias Veterinarias, Universidad de Buenos Aires.

### 2.2. Experimental Design

#### 2.2.1. Phase 1

Seven llamas weighing (mean ± SD) 98.0 ± 19.4 kg were used during this phase. Each one received 10 mg/kg BW of cephalexin lysine aqueous immediate release solution (200 mg/mL Cefalexina Richet® 1 g, Laboratorio Richet, Buenos Aires, Argentina) by the IV, IM, and SC routes in a three-part randomized crossover design with a 2-week washout period between treatments. The IV administrations were injected as a bolus into the right jugular vein, the IM administrations were injected into a bare region of the gluteus, and the SC administrations were injected under a skin fold in a bare region on the lateral of the thorax. Heparinized blood samples (2.5 mL) were collected via left jugular venipuncture at 0.08, 0.16, 0.25, 0.33, 0.5, 0.75, 1, 1.5, 2, 2.5, 3, 4, 5, 6, 8, 10, and 12 h after drug administration. Blood samples were maintained under refrigeration waiting centrifugation at 3000 ×g for 10 min within 2 h of collection. The supernatant plasma was frozen at −20°C until analysis.

#### 2.2.2. Phase 2

Six llamas weighing (mean ± SD) 121.2 ± 21.6 kg were used during this phase. Each one received 8 mg/kg of sustained release 20% cephalexin suspension (Cefalexina Ruminal® 20%, Laboratorio Ruminal, Buenos Aires, Argentina) by the IM and SC routes in a randomized crossover design with a 2-week washout period between treatments. Heparinized blood samples (2.5 mL) were collected via both jugular venipuncture at 0.25, 0.5, 0.75, 1, 1.5, 2, 3, 4, 6, 8, 10, 12, 14, 24, 27, 30, 33, 36, 38, and 48 h after drug administration. Blood samples were treated as described in phase 1.

### 2.3. Analytical Assay

Concentrations of cephalexin in plasma were determined by microbiological bioassay [12] using* Kocuria rhizophila* ATCC 9341 as test microorganism. The standard curve was prepared in normal llama plasma the same day the blood samples were collected. Each sample was plated in triplicate and each standard dilution was repeated four times. The method was linear between 0.39 and 150 *μ*g/mL (*r* = 0.99). The limits of detection and quantification of the method were 0.39 and 0.78 *μ*g/mL, respectively. The limit of quantification was the lower concentration used for the pharmacokinetics analysis. The interassay and intraassay coefficients of variation were <7% and <8%, respectively. Accuracy of the assay ranged between 82 and 99%.

### 2.4. Pharmacokinetic Calculations

Cephalexin concentration-time data in plasma for each animal and each route of administration were analyzed by noncompartmental techniques (PCNONLIN 4.0 Software, SCI Software, Lexington, KY, USA). For peak concentration in plasma (*C*_max_) and time to peak concentration in plasma (*T*_max_) observed values were taken. The apparent terminal rate constant (*λ*) was determined by linear regression of the last 4-5 points on the terminal phase of the logarithmic plasma concentration-time curve. The terminal half-life (*t*_1/2*λ*_) was calculated as ln⁡2/*λ*. The area under the plasma concentration-time curve (AUC) for the time at which the final measurable concentration was obtained (AUC_0–last_) was calculated by the linear trapezoidal rule. The AUC from the final time point to time infinity (AUC_last–*∞*_) was estimated as the ratio of the final observed concentration/*λ*. The total AUC (AUC_0–*∞*_) was calculated by addition of AUC_0–last_ and AUC_last–*∞*_. The mean residence time (MRT) was calculated as AUMC/AUC, where AUMC is the area under the curve of the product of time and the plasma drug concentration-time from time zero to infinity. The mean absorption time (MAT) was calculated as MRT_(IM, SC)_ − MRT_(IV)_. Total body clearance (Cl_t_) was calculated as the ratio of the intravenously administered dose to AUC_0–*∞*_ and the apparent volume of distribution at steady state (*V*_ss_) was estimated as Cl_t_·MRT. Absolute bioavailability (*F*) was calculated as *F* = AUC_0–*∞* (IM, SC)_/AUC_0–*∞* (IV)_ · 100. All values are reported as mean ± standard deviation (SD).

### 2.5. MIC Determination and PK/PD Indices

A total of 11* E. coli* and 9 coagulase-positive staphylococci isolates recovered from adult llamas were tested for cephalexin susceptibility by the broth macrodilution method according to the guidelines of the Clinical and Laboratory Standards Institute [13]. The range of cephalexin tested concentrations was 32–0.25 *μ*g/mL. The MIC_50_ and MIC_90_ were defined as the cephalexin concentrations that inhibit the growth of 50 and 90% of tested isolates, respectively. The quality of the results was determined by concurrent testing of* Staphylococcus aureus* ATCC 29213 and* Escherichia coli* ATCC 25922.

*T* > MIC_90_ was calculated graphically for each animal for the three routes of administration and both formulations and was expressed in hours (mean ± SD) and percentage of the recommended dosing interval.

### 2.6. Statistical Analysis

A computerized program (GraphPad Prism, 5.0, GraphPad Software Inc, San Diego, California, USA) was used to identify the presence of differences between log-transformed parameters [14] calculated after treatments, using repeated measures ANOVA followed by a* post hoc* Tukey's multicomparison test when appropriate, and paired *t*-test. Wilcoxon's matched pairs test was used for *T*_max_ comparison. In addition, significant differences between formulations administered by the same route were detected by a nonpaired *t*-test (log-transformed parameters) and a Mann-Whitney test (*T*_max_). A value of *p* ≤ 0.05 was considered significant.

## 3. Results

Cephalexin was well tolerated in all but one llama, in which following its IV administration a facial edema was observed; thus, this animal was immediately retired from the experience, and six animals were used for pharmacokinetic calculations during phases 1 and 2.

**Figure 1:**
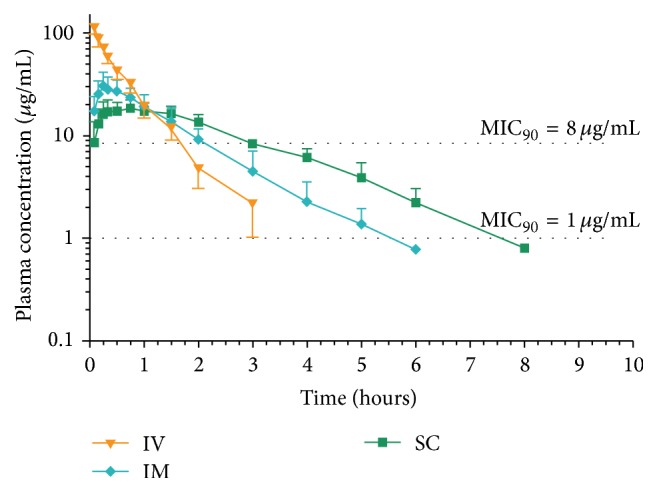
Mean ± SD of plasma cephalexin concentrations following immediate release formulation administration (10 mg/kg) by the intravenous (IV), intramuscular (IM), and subcutaneous (SC) routes to 6 llamas.

**Figure 2:**
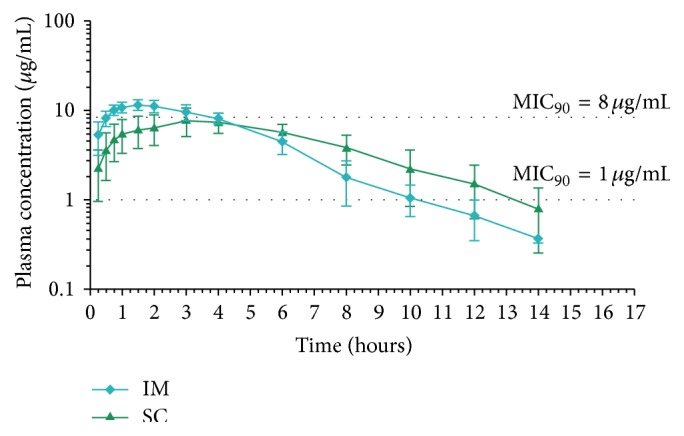
Mean ± SD of plasma cephalexin concentrations following sustained release formulation (8 mg/kg) administration by the intramuscular (IM) and subcutaneous (SC) routes to 6 llamas.

The mean cephalexin plasma concentration-time curves following all treatments are presented in Figures 1 and 2. The pharmacokinetic parameters and statistical analysis calculated for both cephalexin formulations are shown in Tables 1 and 2. The slow absorption of the long-acting formulation was demonstrated by the significantly lower *T*_max_ calculated following the administration of the immediate release cephalexin formulation, by both the IM and the SC (0.36 versus 1.75 h and 0.8 versus 3.3 h, resp.) routes. Terminal half-life was significantly lower for the immediate release formulation when administered by both routes (0.7 versus 1.6 h and 1.3 versus 2.6 h, resp.) when compared with the long-acting formulation. Cephalexin plasma concentrations were detected up to 3, 4–6, and 5–8 h following the IV, IM, and SC administrations, respectively, of the immediate release, and for 6–12 and 8–14 h following the IM and SC administrations, respectively, of the sustained release formulations.

Cephalexin MIC_50_ and MIC_90_ values against coagulase-positive staphylococci were 0.5 *μ*g/mL and 1.0 *μ*g/mL, respectively. Both cephalexin MIC_50_ and MIC_90_ values were 8.0 *μ*g/mL against* E. coli*. MIC values of the quality control strains (*Staphylococcus aureus* ATCC 29213 and* E. coli* ATCC 25922) were within the reference range. *T* > MIC for both cephalexin formulations are shown in Tables 3 and 4. For the immediate release formulation, *T* > MIC values for coagulase-positive staphylococci, calculated as percentage of a 6 and 8 h dosing interval, ranged between 60.6–100.0 and 45.5–88.6%, respectively, whereas, for the long-acting administration, *T* > MIC values were 81.6 and 100.0% for the 12 h dosing interval, and 40.8 and 51.5% for the 24 h dosing interval. *T* > MIC values for* E. coli* for the immediate release formulation ranged between 28.2–55.6 and 21.1–41.7%, for the 6 and 8 h dosing interval, respectively, whereas, for the long-acting administration, *T* > MIC values were 26.2 and 20.1% for the 12 h dosing interval and 13.1 and 10.0% for the 24 h dosing interval, respectively.

## 4. Discussion

In this study we describe and compare the pharmacokinetics of a single administration of immediate and sustained release cephalexin by the IV, IM, and SC routes to six adult healthy llamas, in order to identify data useful for determining a dosage regimen that could provide therapeutic plasma concentrations in this species.

The microbiological assay is useful for determining the plasma concentrations of those antimicrobial agents that are not transformed into active metabolites, as almost all beta-lactams, including cephalexin. The methodology used in this study has been validated in our laboratory and was used for previous cephalexin pharmacokinetic characterizations in goats [11], cattle [10], and dogs [2, 3]. The immediate release cephalexin dosage was chosen according to those used in previous pharmacokinetic studies in ruminant species as cows and goats [7, 8, 10, 11]; meanwhile, the dosage of long-acting cephalexin was the one indicated by the manufacturer for other species (feline, canine, bovine, ovine, and pigs).

Several previous studies have reported pharmacokinetic data for cephalexin in different species, including those frequently used as reference for dose rate extrapolation in llamas, as cows, goats, and sheep [5–11]. However, to our knowledge, the present publication is the first to provide pharmacokinetic data in llamas using two different cephalexin formulations, even though long-acting formulations are frequently used in the field.

### 4.1. Immediate Release Cephalexin Formulation

Noncompartmental analysis of the disposition curves following the administration of the immediate release cephalexin formulation showed that, after IV injection, cephalexin is rapidly eliminated; the drug was detected up to 3 h after administration in the six animals. The half-life calculated in our study (0.60 h) is short, mainly due to a very small volume of distribution (Vd_ss_ 0.102 L/kg), similar to the one reported by Ambros et al. [11] in goats (0.36 h) and slightly lower than the almost 1 h described by other authors in calves [5, 6] and sheep (72 min) [9]. The MRT (0.7 h) in our study was similar to the one reported for goats (0.46 h) [11].

Cephalexin clearance (2.42 mL/min·kg) was lower than the one described in goats (0.35 L/kg·h) [11]. It has been reported that glomerular filtration rate is 1.33 mL/min·kg in camels [15] and 102.6 mL/kg in adult llamas [16]; thus, our data suggest that an active renal secretion in addition to glomerular filtration may occur in this species. A similar suggestion has been made by Villa et al. [4] for cephalexin excretion in horses. Many cephalosporins are excreted into urine via glomerular filtration and active tubular secretion, and it has been reported that a renal organic anion carrier mediates the transport of cephalexin into urine [16, 17]. The small distribution volume (Vd_ss_ 0.102 L/kg) is similar to those previously reported in goats (0.16 L/kg) [11] and calves (0.17 L/kg) [9] but smaller than the one described in calves (0.89 L/kg; 0.45 L/kg) [5, 9], and in agreement with the limited distribution of beta-lactams. With similar doses, AUC values calculated for goats were lower than those calculated in this study (28.80 *μ*g·h/mL) [11].

Following the IV administration, one llama developed a mild hypersensitive reaction, consisting of facial swelling, which could be considered drug related. This condition was successfully treated with a single IM dose (1 mg/kg) of dexamethasone; the animal recovered very quickly and was withdrawn from the experience.

In this study, the extent of cephalexin absorption by both IM and SC routes was similar, as indicated by the lack of difference between AUC and absolute bioavailability (72% and 89%, for the IM and SC routes, resp.), and indicates good absorption of the drug from the site of injection. No significant differences of *C*_max_ and *T*_max_ were found; however, the high variability of IM data may account for this result. MAT_IM_ was significantly lower than MAT_SC_ (0.6 h versus 1.7 h, resp.). The half-life following the IM (0.67 h) administration was significantly lower than that calculated for the SC administration (1.3 h); the longer half-life may be due to a flip-flop phenomenon as a result of the extended absorption in the SC administration, affecting cephalexin elimination [18, 19]. Longer half-lives after IM administration have been described in calves by Garg et al. (2.0 h) [8] and Archimbault et al. (4.6 h) [5]; however, in the latter study an oily cephalexin suspension was administered. Following the IM administration, *C*_max_ was higher than the one reported by Archimbault et al. (7.42 *μ*g/mL) [5]. Using the same single dose, IM bioavailability described by Garg et al. [8] following IM administration (81.9%) was similar to the one reported here.

### 4.2. Long-Acting Cephalexin

A cephalexin long-acting formulation was studied in order to investigate if the delayed absorption allowed an extended dosing interval without affecting the predicted clinical efficacy, thus reducing the cost of treatment, enhancing prescription compliance, and favoring animal welfare. Long-acting formulations are considered more likely to present variability in their absorption than aqueous solutions. Our results showed that significant differences were detected for *T*_max_ (1.75 versus 3.33 h, for the IM and SC administration, resp.). Absolute bioavailability was high for both routes of administration (87 and 84%), and similar to that calculated for the soluble lysine formulation. Waxman Dova et al. [10] reported significantly higher SC half-life values when compared with IM administration (4.2 versus 1.8 h, resp.) in cows receiving a long-acting cephalexin oily formulation. However, the high variability in the half-life values following the SC route in our study may have accounted for the lack of difference between both administrations.

### 4.3. Pharmacokinetic-Pharmacodynamics Modelling

Beta-lactam antibiotics present a time-dependent bactericidal activity, and it has been reported that clinical success results when plasma concentrations exceed MIC against the infecting pathogens for at least 50 and possibly 80% of the dosage interval [1, 20]. Thus, we calculated the *T* > MIC for recommending a rational dosage regimen for cephalexin in llamas. The number of strains obtained from llamas that was used in this study for the MIC determination is low; however, to the authors' knowledge there are no reports on cephalexin MIC in isolates from camelids that could be used for calculating *T* > MIC.

Our results showed that both formulations failed to exceed the MIC_90_ calculated for our* E. coli* strains of llama origin for the required 50–80% of the recommended dosing interval (8–12 and 48 h, for the immediate and sustained release, resp.), suggesting that a higher frequency of administration, a higher dose, or both, compared to those used in this study, would be necessary for a successful clinical outcome. For the clinical treatment of Gram-positive susceptible cocci, a different dosing strategy would be required for the IM or the SC administration, as both provide different bacterial exposure. The immediate release formulation would be effective for treating staphylococcal infections administered every 8 h (IM) or 12 h (SC) at a 10 mg/kg dose level, whereas the sustained release formulation would require the IM or SC administration of a 8 mg/kg dose level every 12 or 24 h, respectively. The IV administration of a 10 mg/kg dose may provide approximately 2 h of surgical prophylaxis against susceptible pathogens and could be repeated if surgical procedures extend beyond that period.

This study has demonstrated that both the route of administration and the pharmaceutical formulation may affect cephalexin pharmacokinetic parameters in llamas and has generated data for recommending a therapeutic dosage regimen; however, possible limitations of this study may be recognized. On the one hand, the calculated *T* > MIC values may be relatively imprecise, due to the small number of strains and the recommended CLSI doubling dilution method. However, the MIC_90_ values against* E. coli* (8 *μ*g/mL) and positive-coagulase staphylococci (1 *μ*g/mL) calculated in this study were similar to those previously reported for strains of bovine origin [21, 22]. It has previously been reported that cephalexin MIC for susceptible bacteria of veterinary origin ranged between 0.25 and 8 *μ*g/mL [23]. On the other hand, healthy well fed animals were used for this study; illness, body conformation, and breeding environment could affect any of the pharmacokinetic phases, modifying plasma cephalexin concentrations. Thus, further clinical studies will be needed to determine the efficacy of cephalexin for treating bacterial infections in this species.

## Figures and Tables

**Table 1:** Pharmacokinetic parameters (mean ± SD) calculated for immediate release cephalexin following an intravenous (IV), intramuscular (IM), and subcutaneous (SC) 10 mg/kg injection to 6 llamas.

Parameter	Route of administration		
	IV	IM	SC
*C*_max_ (*µ*g/mL)	—	31.2 ± 10.5^a^	19.1 ± 3.5^a^
*T*_max_ (h)	—	0.36 ± 0.2^a^	0.84 ± 0.6^a^
*λ*_*z*_ (h^−1^)	1.17 ± 0.18^a^	1.05 ± 0.16^a^	0.55 ± 0.11^b^
*t*_1/2*λ*_ (h)	0.60 ± 0.1^a^	0.67 ± 0.1^a^	1.3 ± 0.2^b^
AUC_0–last_ (*µ*g·h/mL)	67.5 ± 6.0^a^	49.0 ± 11.5^bc^	60.3 ± 5.7^ac^
AUC_0–inf_ (*µ*g·h/mL)	69.5 ± 6.8^a^	49.4 ± 11.4^bc^	61.8 ± 5.7^ac^
MRT_last_ (h)	0.6 ± 0.08^a^	1.3 ± 0.4^b^	2.2 ± 0.4^c^
MRT_inf_ (h)	0.7 ± 0.1^a^	1.4 ± 0.4^b^	2.4 ± 0.5^c^
MAT (h)	—	0.6 ± 0.4^a^	1.7 ± 0.5^b^
Cl_t_ (mL/min·kg)	2.42 ± 0.25	—	—
Cl_t_/*F* (mL/min·kg)	—	3.49 ± 0.6	2.72 ± 0.2
Vd_*z*_ (L/kg)	0.135 ± 0.01^a^	0.280 ± 0.06^b^	0.270 ± 0.05^b^
Vd_ss_ (L/kg)	0.102 ± 0.01	—	—
*F* (%)	—	72 ± 20^a^	89 ± 10^a^

^a.b.c^Values within a row with different superscripts indicate significant differences (*p* < 0.05).

*C*_max_: peak serum concentration; *T*_max_: time to reach peak serum concentration; *λ*: apparent terminal rate constant; *t*_1/2λ_: terminal half-life; AUC_0–last_: area under the serum concentration-time curve from time zero to last point; AUC_0–∞_: area under the serum concentration-time curve from time zero to infinity; MRT: mean residence time; MAT: mean absorption time; Cl_t_: total body clearance; Vd_*z*_: apparent volume of distribution; Vd_ss_: volume of distribution at the steady state; *F*: absolute bioavailability.

**Table 2:** Pharmacokinetic parameters (mean ± SD) calculated for sustained release cephalexin following an intramuscular (IM) and subcutaneous (SC) 8 mg/kg injection to 6 llamas.

Parameter	Route of administration	
	IM	SC
*C*_max_ (*μ*g/mL)	11.7 ± 1.6^a^	8.2 ± 2.4^a^
*T*_max_ (h)	1.75 ± 0.3^a^	3.33 ± 0.5^b^
*λ* (h^−1^)	0.46 ± 0.13^a^	0.31 ± 0.10^a^
*t*_1/2*λ*_ (h)	1.60 ± 0.4^a^	2.65 ± 1.7^a^
AUC_0–last_ (*μ*g·h/mL)	59.5 ± 6.7^a^	57.7 ± 12.3^a^
AUC_0–inf_ (*μ*g·h/mL)	60.2 ± 6.7^a^	57.9 ± 12.4^a^
MRT_last_ (h)	3.5 ± 0.6^a^	5.1 ± 1.2^b^
MRT_inf_ (h)	3.7 ± 0.7^a^	6.1 ± 2.5^a^
*F* (%)	87 ± 10^a^	84 ± 18^a^

^a.b^Values within a row with different superscripts indicate significant differences (*p* < 0.05).

*C*_max_: peak serum concentration; *T*_max_: time to reach peak serum concentration; *λ*: apparent terminal rate constant; *t*_1/2*λ*_: terminal half-life; AUC_0–last_: area under the serum concentration-time curve from time zero to last point; AUC_0–∞_: area under the serum concentration-time curve from time zero to infinity; MRT: mean residence time; *F*: absolute bioavailability.

**Table 3:** Time above MIC_90_ (*T* > MIC) calculated for cephalexin following an intravenous (IV), intramuscular (IM), and subcutaneous (SC) 10 mg/kg injection to 6 llamas. Results (mean ± SD) are expressed in hours (h) and dosing interval (DI) percentage.

*T* > MIC	*Escherichia coli* (8 *µ*g/mL)			Coagulase-positive staphylococci (1 *µ*g/mL)		
	IV	IM	SC	IV	IM	SC
(h)	1.7 ± 0.1	2.3 ± 0.5	3.3 ± 0.3	3.6 ± 0.5	4.4 ± 0.8	7.1 ± 1.2
(% 6 h DI)	28.2	38.5	55.6	60.6	73.0	100.0
(% 8 h DI)	21.1	29.0	41.7	45.5	54.5	88.6

**Table 4:** Time above MIC_90_ (*T* > MIC) calculated for long-acting cephalexin following an intramuscular (IM) and subcutaneous (SC) 8 mg/kg injection to 6 llamas. Results are expressed in hours (h) and dosing interval (DI) percentage.

*T* > MIC	*Escherichia coli* (8 *µ*g/mL)		Coagulase-positive staphylococci (1 *µ*g/mL)	
	IM	SC	IM	SC
(h)	3.1 ± 1.2	2.4 ± 1.5^*∗*^	9.8 ± 1.4	12.4 ± 2.1
(% 12 h DI)	26.2	20.1	81.6	100.0
(% 24 h DI)	13.1	10.0	40.8	51.5

^*∗*^Data of 4 animals.
